# Growth and Survival Variation among Scots Pine (*Pinus sylvestris *L.) Provenances

**DOI:** 10.1155/2017/1904623

**Published:** 2017-01-04

**Authors:** Süleyman Gülcü, Nebi Bilir

**Affiliations:** Forestry Faculty, Suleyman Demirel University, 32260 Isparta, Turkey

## Abstract

Tree height, basal diameter, and survival were examined in thirteen-year-old provenance test established by 30 seed sources of Scots pine (*Pinus sylvestris* L.) at two exotic sites of the species in Southern part of Turkey. Variations within provenance and among provenances and relations among the traits were estimated to compare Scots pine provenance and two other native species. Averages of tree height and basal diameter were 350 cm and 52.7 mm in Aydogmus site and 385 cm and 51.2 mm in Kemer site, respectively. There were large differences within and among provenances for the characters. Sites were similar (*p* > 0.05) for the characters, while there were significant differences (*p* ≤ 0.05) among provenances within site according to results of variance analysis (ANOVA). Scots pine provenances were higher and had more thickness than that of black pine (*Pinus nigra* Arnold) and Taurus cedar (*Cedrus libani* A. Rich.) which were natural species of the region. There were positive and significant (*p* < 0.05) correlations between height and basal diameter in the species. Average survivals were 56% and 35% of the provenances in the sites. They were 71% and 11% in black pine and 53% in Taurus cedar for the sites respectively.

## 1. Introduction

**Figure 1:**
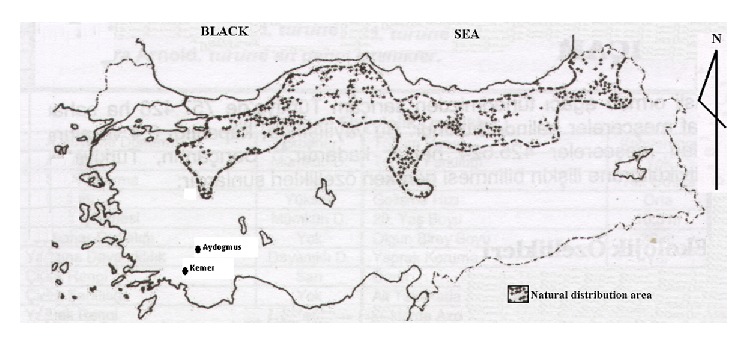
Natural distribution area of Scots pine in Turkey, and experimental sites.

Scots pine (*Pinus sylvestris*) is classified as one of the economically important tree species for Turkish forestry in the “National Tree Breeding and Seed Production Programme” [1]. The species grows between 0 and 2700 meters above sea level in Turkey. Pure and mixed stands of the species occupy roughly 750 000 ha in Turkey, of which roughly 475 000 ha are considered to be productive forests (Figure 1).

Scots pine is one of the valuable commercial forest tree species in Europe and Asia. Besides, it is interesting as an introduced exotic species in Korea, China, Mexico, and New Zealand, where provenance and cultivation trials have been established [2]. Within its wide natural and artificial range in the Euroasiatic continent the species shows large variability of its adaptation and growth features [3]. Provenance trials with forest trees provide valuable information about growth and adaptability of populations often transferred over large geographical and climatic distances [3]. Estimation of provenance variation is also one of the main stages of tree breeding programme, to establish successful plantations and to determine seed transfer regions. Provenance tests with Scots pine date back to as early as the 19th century. Few of those tests would meet the present-day statistical standards required for field experimentation, but frequently the progeny of one stand (provenance) was planted at several experimental sites. Most of those early experiments were limited to the scale of national tests [3]. While many national and international studies were conducted on provenance test for different purposes in the species in many countries (e.g., [4–11]) or different forest tree species (e.g., [12–15]), the present study is one of the first investigations in Southern Turkey which is exotic for the species.

The purposes of this study were to estimate variations of tree height and basal diameter within provenance and among provenances, to compare Scots pine to native black pine (*Pinus nigra*) and Taurus cedar (*Cedrus libani*) species of the sites, and to evaluate relations between height and diameter in the species to estimate better provenance/s for the region.

## 2. Materials and Methods

This study was carried out in two experimental areas (latitude 38°36′N, longitude 30°24′E, and altitude 1100 m asl. called Aydogmus site; latitude 37°35′N, longitude 30°06′E, and altitude 1180 m asl. called Kemer site in the paper) in Southern Turkey, established by 30 Scotch pine provenances and native Taurus cedar and Anatolian black pine provenances for comparison (Table 1).

The experiments were established as “Randomized Blocks” with three blocks and 2.5 × 2 m spacing in year 2000. Each provenance was represented by thirty-two-year-old containerized seedlings in each replication/block. Data of survival, tree height (*H*, cm), and basal diameter (*D*_0_, mm), also called base diameter at soil surface, were collected at thirteen-year-old provenance test in October of 2013. Tree height and basal diameter were measured by Haglöf-Vertex hypsometer and electronic caliper, respectively.

The statistical analysis was carried out by SPSS statistical package according to the following model of ANOVA used for the analysis:

(1) $$\begin{array}{c} Y_{ijkm}=\mu +S_{i}+B_{j\left(i\right)}+P_{k}+SP_{ik}+BP_{j\left(i\right)k}+e_{ijkm}, \end{array}$$

where* Y*_*ijk*_ is the observation from the* m*th tree of* k*th provenance at* j*th block of* i*th site, *μ* is overall mean,* S*_*i*_ is the effect of the* i*th site, *B*_*j*(*i*)_ is the effect of* j*th block at* i*th site,* P*_*k*_ is effect of* k*th provenance,* SP*_*ik*_ is the interaction between* k*th provenance and* i*th site, *BP*_*j*(*i*)*k*_ is interaction between* k*th provenance and* j*th block at* i*th site, and* e*_*ijkm*_ is random error. Provenances were grouped by Duncan's multiple range test. Individual phenotypic correlations among traits were also calculated.

Correlation between tree height and basal diameter was also calculated by Pearson's correlation using SPSS statistical package program [16].

## 3. Results and Discussion

### 3.1. Traits

Survival rate, averages, and ranges of the tree height and basal diameter for provenances and sites were given in Table 2.

**Figure 2:**
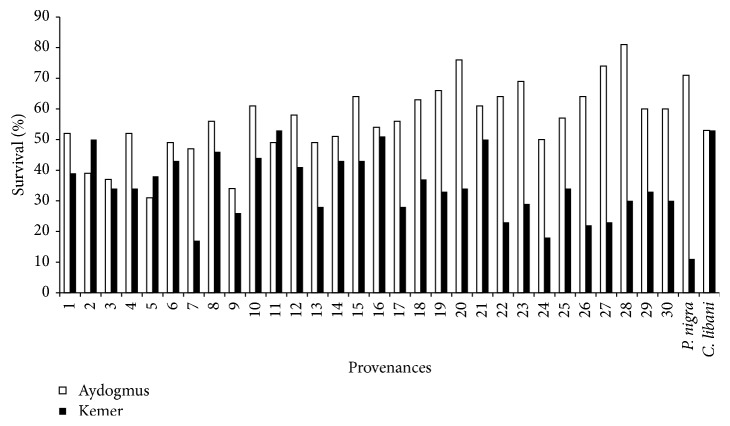
Survival of provenances for site.

Survival was one of the most important criterions in economical and biological success of plantation forestry. It was gaining importance based on climate change by provenance test. It was emphasized that provenance trials series with many test localities covering a large variation of climatic conditions were ideal for estimates of the consequences of changes of temperature climate [9]. Survival rates were changed for species, sites, and provenances (Table 2). They were 56% (varied between 31% and 76%) and 35% (18% and 53%) in experimental sites. While the survival was lowest (53%) for Taurus cedar in first site, it was the highest (53%) for the species in second site. There was no any higher survival provenance of Scots pine than Taurus cedar in second site (Table 2 and Figure 2).* Pinus sylvestris* provenances showed better survival performance than that of* P. contorta* in Sweden [10]. Average survival was reported to be 44.5% with overall Scots pine provenance trials ranging from 5 to 17 years in Poland [17]. It was 41.5% in 30th year results of provenance trials in the species [3]. Large differences among provenances for survival were also reported in provenance trials of Scots pine in different countries [3, 11, 18, 19]. Significant relationship between survival and population/origin was found in provenance trials of Scots pine [20]. It was known that there could be many environmental and genetic effects on survival. The results showed importance of provenance trials for selection of regional seed sources in plantation forestry. In German Scots pine provenance trial results of Taeger et al. [21] highlight the importance of genotype × environment interaction in response to extreme climatic events, which have to be considered in the interpretation of population adaptation to climate change. In Polish provenance trials, results of Barzdajn et al. [3] showed that the southern populations that moved so far north suffered too much of a climatic transfer. On the other hand, good survival of northern populations in a milder environment of our research site, as compared to their native climate, indicates that there was some potential within those populations to adapt to changes in climate corresponding to that transfer.

**Figure 3:**
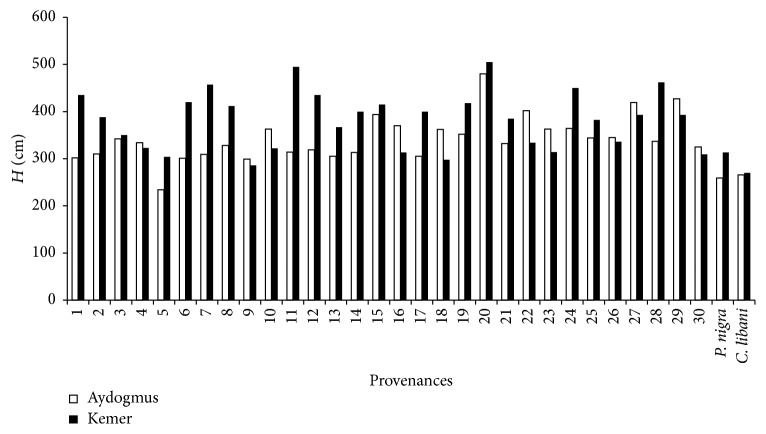
Tree height of provenances for site.

**Figure 4:**
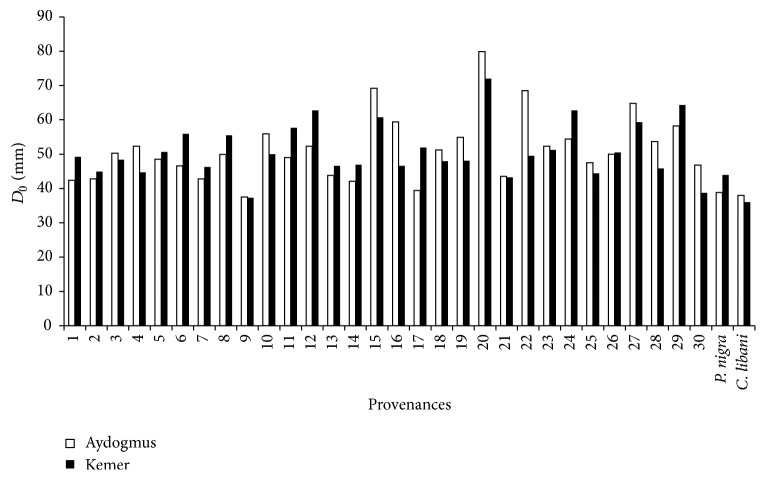
Basal diameter of provenances for site.

Averages of tree height and basal diameter were 350 cm and 385 cm and 52.7 mm and 51.2 mm in the sites, respectively (Table 2). As seen from Table 2, there were large differences within provenance and among provenances for tree height and basal diameter. The result was well in accordance with the early results from the species [3, 11, 19, 22, 23]. These results indicated that there was a large variation among tested populations in growth at the examined test site and within species. However, while there were 15–20% differences for height and radial growth among provenances of Norway spruce, qualitative traits such as stem shape, branch density, and shape and health state were similar in the species [12] and had statistically significant difference (0.05 > *p*) among populations of black pine reported by Gülcü et al. [24] for growth characters. Scots pine provenances showed higher height and diameter performances than that of black pine and Taurus cedar which were natural forest tree species of the sites (Figures 3 and 4) opposite to primary results of the trial [25]. However, growth performances could chance for the provenances in the future as also emphasized in Polish provenance trials of Scots pine by Barzdajn et al. [3] and other trials [25, 26].

Averages of tree height and basal diameter of seed stand provenances were generally higher than that of seed orchard provenances marked in Table 1. This was not an expected situation. However, seed orchards have been established with clones or seedlings, collected from plus trees selected phenotypically from natural seed stands. Besides, it could be said that provenances of the present study had higher height and diameter than early studies [4–6].

Sites were similar for tree height and basal diameter (*p* > 0.05), while there were significant differences (*p* ≤ 0.05) among provenances according to results of analysis of variance (ANOVA) (Table 3) and Duncan's multiple range test (Table 4). However, site × provenance interaction was significant (*p* < 0.05). The result was well in accordance with the early results from provenance trials of the species [3, 19]. Ulbrichová et al. [12] reported that environmental variables were significantly effective on growth characters in provenance test of Norway spruce. Significant site × provenance interaction was reported in a provenance test of* Eucalyptus robusta* [15]. The interaction result of the present study showed large variation for selection capacity and adaptation ability to different site of the species.

### 3.2. Correlations among the Traits

Positive and significant (*p* ≤ 0.05) correlations were found between tree height and basal diameter in Scots pine (*r* = 0.643), in black pine (*r* = 0.405), and in Taurus cedar (*r* = 0.634). Positive and significant correlation was reported between height and growth traits in Scots pine populations [3, 5, 11] and between tree height and branch diameter in black pine populations [24] and also* Eucalyptus urophylla* provenances [14]. The relationship could be used in the future studies on the species.

## 4. Conclusions

In this paper, we reported significant variation in the growth traits and survival among 30 Scots pine provenances and two other native species based on thirteen-year-old provenance trials in Southern Turkey. The variation among provenances and within provenance emphasized importance of mass and individual selection and large adaptation ability of the species. There were positive and significant correlations between growth traits. The relationship could be used in forestry practices of the species such as pruning. The phenotypic selection based on growth traits should be combined by genotypic selection in the future studies.

Scots pine provenances were higher and had more thickness than that of black pine and Taurus cedar. It showed that the Scots pine could be used at low ratio (less than 10%) in mixed plantation at the region. Therefore, it was needed to collect more data to draw accurate suggestion for better provenance for the region.

## Figures and Tables

**Table 1:** Geographic details of the provenances.

Provenance number	Country	Latitude (N)	Longitude (E)	Altitude (m)
1	Turkey	40°53′	3220	1550
2	Turkey	40°38′	4228	2050
3 (GR)^*∗*^	Greece	41°17′	Unknown	1600
4	Turkey	41°10′	3505	1200
5	Turkey	40°31′	3208	1550
6^*∗∗*^	Turkey	39°58′	3107	1550
7^*∗∗*^	Turkey	40°32′	3209	1575
8	Turkey	41°22′	3320	1250
9	Turkey	40°15′	4240	2300
10^*∗∗*^	Turkey	39°41′	3550	910
11^*∗∗*^	Turkey	39°51′	3106	1320
12	Turkey	38°54′	3110	1675
13	Turkey	39°34′	3552	1750
14	Turkey	40°45′	4233	2250
15	Turkey	41°34′	3122	1300
16	Turkey	40°23′	3755	1950
17	Turkey	40°37′	3139	1350
18	Turkey	41°01′	3421	1600
19	Turkey	39°58′	3109	1550
20	Turkey	41°10′	3503	1300
21	Turkey	40°22′	3752	1650
22	Turkey	41°02′	3337	1500
23	Turkey	39°34′	3260	1800
24 (GR)	Greece	Unknown	Unknown	Unknown
25	Turkey	40°18′	4237	2350
26	Turkey	40°26′	4235	2250
27	Turkey	40°37′	3050	1450
28^*∗∗*^	Turkey	39°45′	3110	1350
29 (FR)	France	45°18′	Unknown	860
30^*∗∗*^	Turkey	39°54′	4118	1570
*P. nigra*	Turkey	37°29′	3043	1000
*C. libani*	Turkey	37°44′	3052	1567

^*∗*^GR: Greece; FR: France. ^*∗∗*^Seed orchards.

**Table 2:** Survival (%), averages, and ranges of tree height (*H*) and basal diameter (*D*_0_) of the provenances in the sites.

Provenance number	Aydogmus site					Kemer site				
	Survival	*H*		*D*_0_		Survival	*H*		*D*_0_	
		Average	Range	Average	Range		Average	Range	Average	Range
1	52	302	144–555	42.4	18.1–70.1	39	435	115–630	49.2	14.2–74.3
2	39	310	129–599	42.8	12.2–97.2	50	388	72–706	44.9	13.2–68.6
3^*∗*^	37	342	97–514	50.3	11.7–79.3	34	350	133–673	48.4	22.1–85.8
4	52	334	121–540	52.3	13.4–108.6	34	323	119–537	44.7	20.0–70.0
5	31	234	84–399	48.5	21.8–78.9	38	304	145–427	50.6	29.4–78.4
6^*∗*^	49	301	112–498	46.6	15.2–78.4	43	420	206–650	55.9	32.8–90.7
7^*∗*^	47	309	118–623	42.8	13.1–88.3	17	457	194–860	46.3	18.9–91.0
8	56	328	139–535	49.9	26.7–109.2	46	412	170–656	55.5	29.7–90.5
9	34	299	81–527	37.5	14.2–67.6	26	286	167–458	37.3	22.8–59.3
10^*∗*^	61	363	148–595	55.9	14.7–107.2	44	322	114–616	50.0	21.0–95.2
11^*∗*^	49	314	124–550	49.0	20.4–93.6	53	495	189–731	57.7	31.0–99.2
12	58	319	157–493	52.3	11.9–99	41	435	203–741	62.7	32.6–97.9
13	49	305	130–494	43.8	16.2–80.0	28	367	144–668	46.6	26.4–88.8
14	51	313	153–525	42.1	16.7–106.6	43	400	156–601	46.9	23.0–74.8
15	64	394	167–699	69.2	31.6–129.6	43	415	68–731	60.7	28.0–91.0
16	54	370	168–533	59.4	23.2–103	51	313	108–606	46.6	71.4–23.8
17	56	305	83–505	39.4	12.4–81.8	28	400	210–687	51.9	30.2–80.6
18	63	362	156–582	51.2	14.6–87.8	37	298	70–440	47.9	18.2–67.6
19	66	352	172–518	54.9	17.3–90.9	33	418	190–578	48.1	22.0–69.5
20	76	480	180–792	79.9	27.8–154.9	34	505	150–851	72.0	31.5–120.0
21	61	332	151–506	43.5	17.4–100.3	50	385	193–590	43.2	25.2–74.4
22	64	402	165–606	68.5	19.3–122.4	23	334	87–543	49.5	26.6–70.0
23	69	363	184–580	52.3	20.5–92.2	29	314	152–477	51.2	31.1–81.0
24	50	364	160–655	54.4	20.2–122.4	18	450	84–746	62.7	18.0–100.5
25	57	344	158–545	47.5	15–82.8	34	382	140–603	44.4	30.9–69.0
26	64	345	114–639	50.0	22.9–90.2	22	336	130–508	50.5	24.5–69.4
27	74	419	151–714	64.8	27.7–116.2	23	393	201–614	59.3	36.7–108.8
28^*∗*^	81	337	150–599	53.7	19–95.2	30	462	198–866	45.8	18.6–75.4
29	60	427	119–795	58.2	20.9–146.2	33	393	128–659	64.3	34.0–102.0
30^*∗*^	60	325	94–508	46.8	20–74.4	30	309	110–519	38.7	18.4–63.3
*Total*	*56*	*349.7*	*81–795*	*52.7*	*11.7–154.9*	*35*	*385*	*81–795*	*51.2*	*13.2–120.0*
*P. nigra*	71	259	107–500	38.8	12.5–67.3	11	313	225–425	43.9	25.2–60.0
*C. libani*	53	266	129–482	38.0	15–78.8	53	270	76–549	36.0	12.0–62.4

^*∗*^Seed orchards.

**Table 3:** Results of analysis of variance for the characters.

Source of	H		D_0_	
variation^*∗*^	*F* value	*p* value^*∗∗*^	*F* value	*p* value
*S*_*i*_	56.070	*p* > 0.05^ns^	0.007	*p* > 0.05^ns^
*B*_*j*_	16.877	*p* > 0.05^ns^	59.829	*p* > 0.05^ns^
*P*_*k*_	8.454	0.05 > *p*	13.034	0.05 > *p*
*SP*_*ik*_	7.637	0.05 > *p*	4.034	0.05 > *p*
*BP*_*j*(*i*)*k*_	2.879	0.05 > *p*	2.928	0.05 > *p*

^*∗*^ *S*_*i*_ is the effect of the *i*th site, *B*_*j*(*i*)_ is the effect of *j*th block at *i*th site, *P*_*k*_ is the effect of *k*th provenance, *SP*_*ik*_ is the interaction between *k*th provenance and *i*th site, and *BP*_*j*(*i*)*k*_ is the interaction between *k*th provenance and *j*th block at *i*th site; ^ns^difference is not statistically significant.

**Table 4:** Homogenous groups of Duncan's multiple range tests.

*H*		*D*_0_	
Provenance number	Homogenous groups^*∗*^	Provenance number	Homogenous groups
*P. nigra*	a	*C. libani*	a
*C. libani*	a	9	a
5	a	*P. nigra*	ab
9	ab	21	bc
30	bc	17	bcd
13	cd	7	bcd
4	cde	2	bcde
17	cdef	30	bcdef
18	cdef	14	bcdef
16	cdef	13	bcdef
26	cdefg	1	bcdef
3	cdefgh	25	bcdefgh
10	cdefgh	4	cdefh
7	cdefgh	3	cdefghi
23	cdefgh	5	cdefghi
14	cdefgh	18	cdefghi
2	cdefgh	26	defghi
21	cdefgh	6	cdefghi
6	cdefgh	28	fghi
25	cdefgh	16	hij
1	defgh	23	hij
8	defghi	8	ij
12	efghi	19	ij
28	fghi	10	ij
19	ghij	11	ij
22	ghijk	24	jk
24	hijk	12	jk
15	ijk	29	kl
11	jk	22	l
27	k	27	l
29	k	15	l
20	k	20	m

^*∗*^The same letters are significantly different (*p* > 0.05). ^*∗∗*^Seed orchards.

## References

[1] Koski V., Antola J. (1993). *National Tree Breeding and Seed Production Programme for Turkey 1994–2003*.

[2] Boratynski A., Grietchy M., Mátyás C. (1991). Range of natural distribution. *Genetics of Scots Pine*.

[3] Barzdajn W., Kowalkowski W., Chmura D. J. (2016). Variation in growth and survival among European provenances of Pinus sylvestris in a 30-year-old experiment. *Dendrobiology*.

[4] Saatcioglu F. (1967). Results of the 25 years' provenance experiment established by using 16 Scotch pine of European and 1 of native provenances in Turkey. *Silvae Genetica*.

[5] Stephan B. R., Liesebach M. (1996). Results of the IUFRO 1982 Scots pine (Pinus sylvestris L.) provenance experiment in Southwestern Germany. *Silvae Genetica*.

[6] Dagdas S., Tosun S., Atasoy H., Dasdemir I. (1997). The first preliminary results of scotch pine (*Pinus sylvestris* L.) provenances tests in Turkey. *Technical Bulletin*.

[7] Shutyaev A. M., Giertych M. (1997). Height growth variation in a comprehensive Euroasian provenance experiment of *Pinus sylvestris* L.. *Silvae Genetica*.

[8] Persson B., Ståhl E. G. (1990). Survival and yield of *Pinus sylvestris* L. as related to provenance transfer and spacing at high altitudes in northern Sweden. *Scandinavian Journal of Forest Research*.

[9] Ståhl E. G. (1998). Changes in wood and stem properties of *Pinus sylvestris* caused by provenance transfer. *Silva Fennica*.

[10] Ericsson T. Survival of *Pinus contorta* and *Pinus sylvestris* in northern Sweden.

[11] Gulcu S., Bilir N. (2015). Provenance variations of scots pine (*Pinus sylvestris* L.) in the Southern part of Turkey. *Pakistan Journal of Botany*.

[12] Ulbrichová I., Podrázský V., Beran F. (2015). *Picea abies* provenance test in the Czech Republic after 36 years—central European provenances. *Journal of Forest Science*.

[13] Johnsen K., Creighton H., Jerre L., Maier L., Chris A. Longleaf pine grown in Virginia: a provenance test.

[14] Hodge G. R., Dvorak W. S. (2015). Provenance variation and within-provenance genetic parameters in Eucalyptus urophylla across 125 test sites in Brazil, Colombia, Mexico, South Africa and Venezuela. *Tree Genetics and Genomes*.

[15] Razafimahatratra A. R., Ramananantoandro T., Razafimaharo V., Chaix G. (2016). Provenance and progeny performances and genotype × environment interactions of *Eucalyptus robusta* grown in Madagascar. *Tree Genetics & Genomes*.

[16] Ozdamar K. (1999). *Statistical Analysis by Package Programs*.

[17] Shutyaev A. M., Giertych M. (2003). *Scots Pine (Pinus sylestris L.) in Eurasia—A Map Album of Provenance Site Interactions*.

[18] Beuker E., Valtonen E., Repo T. (1998). Seasonal variation in the frost hardiness of Scots pine and Norway spruce in old provenance experiments in Finland. *Forest Ecology and Management*.

[19] Alía R., Moro-Serrano J., Notivol E. (2001). Genetic variability of Scots pine (*Pinus sylvestris*) provenances in Spain: growth traits and survival. *Silva Fennica*.

[20] Mikola J. (1993). *Provenance and Individual Variation in Climatic Hardiness of Scots Pine in Northern Finland*.

[21] Taeger S., Zang C., Liesebach M., Schneck V., Menzel A. (2013). Impact of climate and drought events on the growth of Scots pine (*Pinus sylvestris* L.) provenances. *Forest Ecology and Management*.

[22] Perks M. P., Mckay H. M. (1997). Morphological and physiological differences in Scots pine seedlings of six seed origins. *Forestry*.

[23] Dutkuner I., Bilir N., Ulusan M. D. (2008). Influence of growth on reproductive traits and its effect on fertility and gene diversity in a clonal seed orchard of scots pine, Pinus Sylvestris L.. *Journal of Environmental Biology*.

[24] Gülcü S., Akcakaya M., Bilir N. (2016). Genetic variation in Anatolian black pine (*Pinus nigra* Arn. subsp. *pallasiana* (Lamb.) Holmboe.) populations. *Journal of Environmental Biology*.

[25] Gezer A., Gülcü S., Bilir N. (2002). Provenance trials of Scots pine (*Pinus silvestris* L.) in Lake district region. *Journal of Forestry Faculty of Suleyman Demirel University*.

[26] Gezer A., Bilir N., Gülcü S. Quality classification of Scots pine (*Pinus silvestris* L.).

